# The Resignation of Dr. Rochester

**Published:** 1893-11

**Authors:** 


### THE RESIGNATION OF DR. ROCHESTER.

Dr. Delancey Rochester, one of the associate editors of this journal, tendered his resignation in that capacity, to take effect January 1, 1892. Upon our request he has deferred an insistence upon its acceptance until this time, but now asks us to drop his name as a member of the Journal staff. We do so with great reluctance, but recognize the fact that Dr. Rochester's large and increasing practice makes such strenuous demands upon his time and strength, as to preclude his further services as one of the editors of the Journal. We hope, however, to publish from time to time contributions from his pen that are always valuable and of interest.

